# Experimental Analysis of Laser Micromachining of Microchannels in Common Microfluidic Substrates

**DOI:** 10.3390/mi12020138

**Published:** 2021-01-28

**Authors:** Prashanth Reddy Konari, Yung-Dai Clayton, Melville B. Vaughan, Morshed Khandaker, Mohammad Robiul Hossan

**Affiliations:** 1 Department of Engineering and Physics, University of Central Oklahoma, Edmond, OK 73034, USA; pkonari@uco.edu (P.R.K.); yteng2@uco.edu (Y.-D.C.); mkhandaker@uco.edu (M.K.); 2 Center for Interdisciplinary Biomedical Education and Research, University of Central Oklahoma, Edmond, OK 73034, USA; mvaughan4@uco.edu; 3 Department of Biology, University of Central Oklahoma, Edmond, OK 73034, USA

**Keywords:** laser ablation, microfluidics, CO_2_ laser micromachining, microfabrication on PDMS, PMMA and Glass, Parametric experimental analysis

## Abstract

Laser micromachining technique offers a promising alternative method for rapid production of microfluidic devices. However, the effect of process parameters on the channel geometry and quality of channels on common microfluidic substrates has not been fully understood yet. In this research, we studied the effect of laser system parameters on the microchannel characteristics of Polydimethylsiloxane (PDMS), polymethyl methacrylate (PMMA), and microscope glass substrate—three most widely used materials for microchannels. We also conducted a cell adhesion experiment using normal human dermal fibroblasts on laser-machined microchannels on these substrates. A commercial CO_2_ laser system consisting of a 45W laser tube, circulating water loop within the laser tube and air cooling of the substrate was used for machining microchannels in PDMS, PMMA and glass. Four laser system parameters—speed, power, focal distance, and number of passes were varied to fabricate straight microchannels. The channel characteristics such as depth, width, and shape were measured using a scanning electron microscope (SEM) and a 3D profilometer. The results show that higher speed produces lower depth while higher laser power produces deeper channels regardless of the substrate materials. Unfocused laser machining produces wider but shallower channels. For the same speed and power, PDMS channels were the widest while PMMA channels were the deepest. Results also showed that the profiles of microchannels can be controlled by increasing the number of passes. With an increased number of passes, both glass and PDMS produced uniform, wider, and more circular channels; in contrast, PMMA channels were sharper at the bottom and skewed. In rapid cell adhesion experiments, PDMS and glass microchannels performed better than PMMA microchannels. This study can serve as a quick reference in material-specific laser-based microchannel fabrications.

## 1. Introduction

Microfluidics has become one of the fastest growing technologies that finds its application in various engineering, biomedical, chemistry, pharmaceutical, biologic, and forensic science applications [1,2]. Microfluidics devices offer numerous competitive advantages over conventional processes such as small physical footprint, reduced reagent consumption, rapid outcomes, and higher efficiency [3,4]. Performance and applications of microfluidic devices depend on the substrate materials and their properties [5,6]. For instance, biological experiments with cells in microfluidics devices need a biocompatible substrate otherwise cells might not be able to survive and perform regular cellular functions [3]. On the other hand, chemical experiments in microfluidics require a chemically stable substrate that can withstand acid, alkali, solvent, and organic reactions [7]. Numerous materials have been proposed and demonstrated to make microfluidic devices such as polydimethylsiloxane (PDMS), poly(methyl methacrylate) (PMMA), silicon and glass, cyclic-olefin-copolymer (COC), polycarbonate (PC), polyethylene terephthalate (PET), and even ceramics [8]. However, PDMS, glass and PMMA are the most common materials that are frequently used to make microfluidic devices. 

Microfluidics devices are generally designed and developed using soft lithography, dry and wet etching, hot embossing and photolithography [9]. Substrate material properties are not only important for a specific application but also for selecting appropriate fabrication techniques. Hot embossing can be used to fabricate PMMA microchannels but not glass or PDMS microchannels. Hence microfluidic researchers are required to have various microfabrication equipment and to learn fabrication processes. However, most of these conventional microfabrication techniques are expensive due to high capital and maintenance costs, are time-consuming, cumbersome, and require trained personnel [10]. Recently laser machining has become a promising technique for fabrication of microfluidic devices [11,12]. Laser machining involves affordability, faster processing time, greater flexibility and more efficiency, lesser residual waste and does not require a cleanroom or trained personnel [13,14]. 

The laser machining process uses an intensely focused beam to break down a material [14]. There are two types of lasers used in microfluidics depending upon the application: pulsed ultraviolet (UV) lasers, or continuous infrared (IR) lasers. Use of high energy light in UV lasers induces breakdown of targeted material in short pulses [15]. This sort of laser has been utilized to add un-moldable features to microchannels [16,17]. However, UV lasers are not often used in fabricating microfluidic devices because UV laser machining systems are generally expensive, complex, and less reproducible. On the other hand, IR lasers (mainly in the form of the CO_2_ laser) are more popular in processing industrial materials including metals and polymers [17,18]. They are commercially available at a cheaper price and more flexible when compared to those of UV lasers. Nevertheless, there are only a handful of reports on the use of CO_2_ laser micromachining of microfluidic devices [11,19,20].

Klank et al. [21] first demonstrated the use of an industrial CO_2_ laser as an effective alternative method to fabricate microfluidic devices in a PMMA substrate. Cheng et. al. [22] proposed thermal annealing of the laser-fabricated PMMA microchannel to reduce the surface roughness of the channel. They also reported that surface roughness and bulge formation along the edge of the laser microchannel depends on the material properties of PMMA (i.e., cast or extruded PMMA). Similar post-machining surface treatment such as chemical bathing [23], thermal processing, or masking with PDMS or copper layer during laser machining [24] and underwater laser machining [24,25] were suggested in the literature for desired channel profile, width, depth, and surface quality. In another report, it was demonstrated that the surface-finish quality of laser-machined PMMA microchannels can be improved by controlling the distance between focal lens and substrate [26]. Prakash and Kumar developed an analytical model to correlate the channel surface quality with the number of passes [24]. Recently our computer simulation studies also showed that the microchannel characteristics such as width, depth and profile shape can be precisely controlled by modulating laser system parameters such as laser power, focal distance, scanning speed and number of passes [27]. 

Although there are handful of studies on the laser machining of PMMA microchannels, reports on the laser machining of PDMS and glass microchannels are very limited, especially for the CO_2_ laser. Darvishi et al [28] demonstrated tapered-shaped microchannels in glass and PDMS using an ultra-fast femtosecond laser with multiple passes. Fu et al. [29] reported the use of a CO_2_ laser-fabricated glass microchannel to detect five Chinese herbs. Their study showed that a second pass of laser machining improves the surface quality and channel profile. In another report, multilayer PDMS devices were fabricated using laser machining [20]. The reported studies mentioned above either were focused on one specific substrate material or studied a few specific laser system parameters. However, microfluidic researchers need to use appropriate substrate materials depending on the sensitivity, precision, and applications of the intended device. 

In addition to the limited reports on laser machined microchannels on common microfluidic substrates, there is no comprehensive experimental analysis or guidelines about how to select appropriate application- and material-specific appropriate laser system parameters to efficiently fabricate microfluidic devices. Hence our goal was to perform a comprehensive experimental analysis using a commercial CO_2_ laser-machining system to understand the impact of laser system parameters such as laser power, focal distance, scanning speed and number of passes on fabricated microchannel characteristics such as width, depth and profile for three most common microfluidic substrate materials: glass, PDMS and PMMA. In addition, a rapid cell adhesion experiment was performed to suggest substrate choices for biological applications of laser machined microchannels. Thus, this parametric experimental investigation can serve as a quick reference for material-specific microchannel fabrications. 

## 2. Materials and Methods 

### 2.1. Substrate Preparation for Micromachining 

Microscopic glass slides (Home Science Tools, Billings, MT, USA) of 75 mm × 25 mm × 1 mm were used as a specimen for glass microchannels. A UV-transparent PMMA sheet of 12-inch square size with about 2.1 mm of thickness was purchased from Nationwide Plastic, Inc (Dallas, TX, USA). The PMMA sheet was then cut into 75 mm × 25 mm pieces. PDMS with curing agent was purchased from Ellsworth (Dow SYLGARD™ 184 Silicone Encapsulant Clear 0.5 kg Kit, Ellsworth, Germantown, WI, USA). PDMS elastomer base and curing agent were mixed at a 1:10 ratio in a disposable mixing boat and put inside a desiccator to remove air bubbles. The elastomer mixture was then poured into a flat glass petri-dish up to a height of 2 mm. The petri-dish was then kept in a hot vacuum chamber with a temperate of 60 °C for 3 h. The cured PDMS was cut into 75 mm × 25 mm pieces and used to create laser machined microchannels on it. 

### 2.2. Experimental Setup

**Figure 1:**
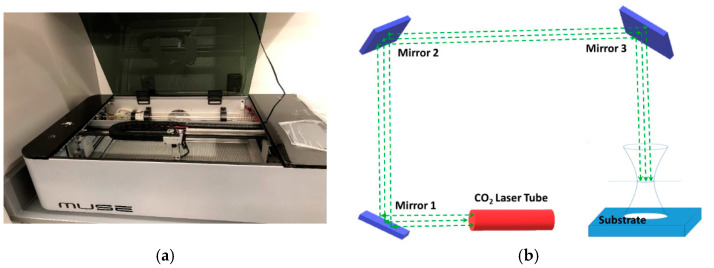
(**a**) Muse commercial CO_2_ laser system; (**b**) Schematics of laser beam paths and position of mirrors.

A commercial 45-watt CO_2_ laser system (Muse Laser System, Full Spectrum Laser, Las Vegas, NV, USA) was used to fabricate microchannels on glass, PDMS and PMMA. The laser system as shown in Figure 1a consisted of an exhaust system, cooling system, and air compressor. The system has three degrees of freedom and a focal lens that can move in x-, y-, and z- directions. The laser beam upon leaving the tube falls on mirror 1, reflects to mirror two, and falls onto mirror 3 as shown in Figure 1b. The laser beam falling on mirror 3 is directed to the object. The system was calibrated by adjusting mirrors prior to microchannel fabrications. The microchannel was drawn in AutoCAD software (Version 23.0, Autodesk, Inc., San Rafael, CA, USA) and was exported to the laser system software (Retina Engrave, Full Spectrum Laser, Las Vegas, NV, USA). Muse software used the run perimeter command to find the laser-firing perimeter. Once the perimeter was found, the substrate was placed inside the perimeter. A microchannel was then created based on the exported AutoCAD file by performing the “Run” command of the laser system.

### 2.3. Characterization Protocols

All specimens after microchannel fabrication were cleaned and washed with soapy water. A scanning electron microscope (Hitachi, Tokyo, Japan) was used to measure the width as well as the depth of the channel. Images were taken under 5 KV and 10 KV accelerating voltages. Channel profile, depth and width of glass microchannels were measured using Profilm 3D profilometer (Filmetrics, San Diego, CA, USA). Profilm3D consisted of white light interferometry (WLI) which was used to measure surface profiles with a precision of 0.05 µm. It had a PSI (phase scanning interferometry) option to take the minimum vertical estimate size down to 0.001 µm.

### 2.4. Sample Preparation for Cell Adhesion Tests

Glass, PDMS and PMMA samples of about 8 mm by 5 mm with laser machined microchannels were prepared. The samples were washed with soapy water, isopropyl alcohol (IPA) and exposed under UV light for an hour. The samples were placed in 24-well plates with the microchannels facing upward. Normal human dermal fibroblasts (HDF00703; LifeLine Cell Technology, https://www.lifelinecelltech.com/) were kept in log-phase growth and cultured/passaged as per standard procedure [30]. Briefly, cells were cultured in a 5% CO_2_ incubator (NuAire 5500), high humidity, at 37 degrees C. Cells were kept in log-phase growth using DMEM/high glucose (Gibco) + 5% FBS (Bio-West) with penicillin/streptomycin/amphotericin (Millipore Sigma). The cells were added into the well plates with media (7 × 10^5^/mL) to cover the surface of the microchannel samples with a cell density of about 9700 cells/cm^2^. The well plates were placed into the incubator for its own incubation time as labeled. After the incubation periods, cells were fixed using methanol-free 4% paraformaldehyde in 0.1M phosphate buffer. The samples were washed twice with PBS and treated with 0.05M Tris Buffer for 30 min to quench unreacted aldehydes. Cells were permeabilized using 0.5% Triton-x-100 for 10 min followed by PBS washes. The samples were stained with Hoechst 33342 stain (Invitrogen, Carlsbad, CA, USA) for 15 min and washed twice with PBS. The samples were mounted in 80% Glycerol in PBS and stored in the freezer. Next day, images were taken for each sample using an inverted fluorescent microscope (IX-71; Olympus, www.olympusamerica.com), and captured using CelSens software. Cells were counted to quantify cell adhesion densities in number of cells/mm^2^. 

### 2.5. Statistical Analysis

All experiments and measurements were done at least 3 times for repeatability and reliability. The results were statistically analyzed by one-way analysis of variance (ANOVA) test using KaleidaGraph (Version 4.5, Synergy Software, Reading, PA, USA). A *p* value of <0.05 was considered statistically significant. The general standard error of the measurement ranged between 0% and 5%. 

## 3. Results and Discussions

The experimental investigation was conducted on the substrates by varying one laser parameter at a time while keeping other parameters unchanged to elucidate the impact of each parameter. For instance, when laser power was varying, the scanning speed, focal distance, and number of passes remained unchanged. The ranges of the varying parameters were based on various literature reviews [20,21,28]. The fabricated channel characteristics (i.e., channel depth, width and profile) were measured after each experiment based on the protocols mentioned above. However, since results among PMMA, PDMS and Glass varied widely, the error bar was not visible in the plot. Hence the results are presented with their average values without showing error bars. For instance, the Table 1 shows variation and standard error calculation of microchannel depth in studying the effect of distances between the specimen and the focusing lens. The detailed restuls and discussions are provided in the following sections. 

### 3.1. Effect of Distances between the Specimen and the Focusing Lens

**Figure 2:**
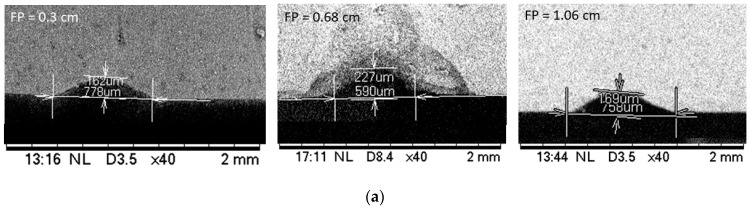

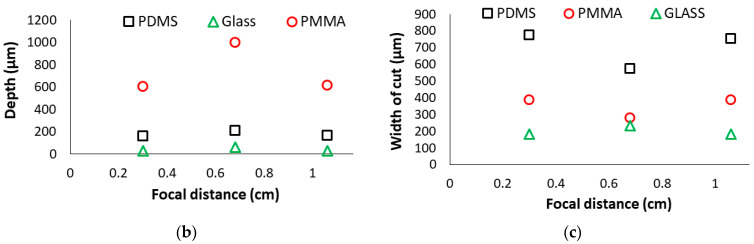
The effect of focal distance on the microchannel characteristics (**a**) SEM measurement of PDMS microchannel at three different focal distances, (**b**) depth of the channel, and (**c**) width of the channel.

A beam is said to be focused when an object is placed exactly at the focal distance from the focusing lens. The fabricated microchannel characteristics (i.e., depth, width and profile) depend on the focused and unfocused laser beams [31]. The effect of the distances between the specimen and focal point of the laser lens is shown in Figure 2. The focal point of the lens used in the laser system was 0.68 cm. The beams were focused when the specimens were placed at this distance from the focusing lens. Specimens were also placed at 0.3 cm and 1.06 cm distance from the lens to study the effect of distance on the microchannel fabrication. Other laser parameters such as scanning speed at 10 mm/s, laser power of 9 W (20% of total power) and number of passes at 1 remained unchanged. 

The specimen SEM images for the PDMS channel are shown in Figure 2a for varying distances between the substrate and focal point of the lens. Figure 2b shows that the cut depth was maximum at the focal distance regardless of the substrate materials. The depth of the channel gradually decreased when the specimens were placed away from the focal point in either direction. At this position of the target substrate, PMMA had a maximum depth of 1000 micrometers followed by PDMS with 162 µm and by glass with 28.3 µm. By contrast, the width of the channel increased as the specimens were placed away from the focal distance in either direction as shown in Figure 2c. When the specimens were placed at the focal length, the laser energy became concentrated around the focal point, thus it penetrated deeper and the spot size became the smallest. Hence, the depth of channels reached maximum and width became minimum which is not counterintuitive. In other words, the laser beam scattered as it was moving away from the focal point and the laser energy intensity became weaker. Since it scattered and became weaker the cut depth decreased and cut width increased. Our previous studies and other reports also confirm this intuitive observation. However, for the glass substrate, it was found that the width increased slightly at the focal distance (Figure 2c). After careful observation of the microchannel surface, we concluded that this can be attributed to the broken edges of the microchannel in the glass substrate. Broken edges in laser-machined glass channels were also reported in previous studies [32]. A simple estimate of the removed volume based on the depth, width and channel profile shows that the approximate material removal rate for PMMA, PDMS and glass were 1.39 mm^3^/s, 0.81 mm^3^/s and 0.097 mm^3^/s respectively for CO_2_ laser power of 9 W and substrates placed at the focal point. It is also interesting to note that PDMS had the greatest cut width while PMMA had the greatest cut depth for the same conditions. The possible explanation for the wider cut in PDMS is that cured PDMS starts decomposing before melting [33] and, thermal conductivity of the cured PDMS is lower than that of PMMA [34]. Cured PDMS starts decomposing at 250 °C [33] and heat does not penetrate deeper due to lower thermal conductivity [27]. In all cases, the glass substrate demonstrated narrower and shallower microchannels for similar laser conditions. This was due to the higher melting temperature of glass. The experimental observation also showed that both the depth and width of the channel matched when the specimens were equidistant from the focal point in either direction (i.e., converging and diverging beams). Nonetheless, the depth and width of a laser-machined microchannel can be controlled by modulating the distance between the target specimen and focal lens.

### 3.2. Effect of Laser Power

**Figure 3:**
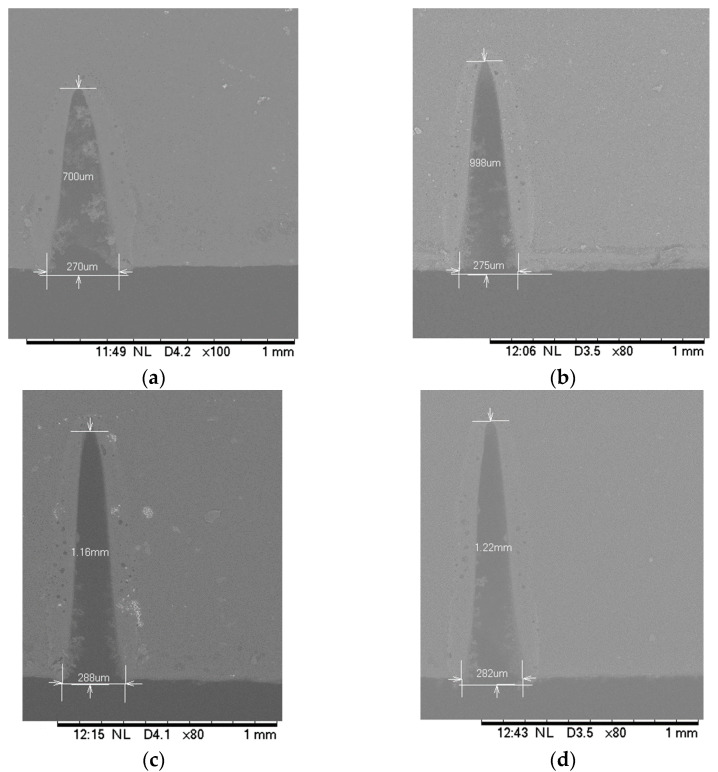
Scanning electron microscope (SEM) images and measurement of CO_2_ laser-machined microchannels on PMMA substrate with (**a**) 10 %, (**b**) 20%, (**c**) 30%, and (**d**) 40% of total laser power. The 45W laser engraving machine was set at single pass and 10 mm/s scanning speed. The PMMA substrates were placed at the focal distance of the laser focusing lens.

**Figure 4:**
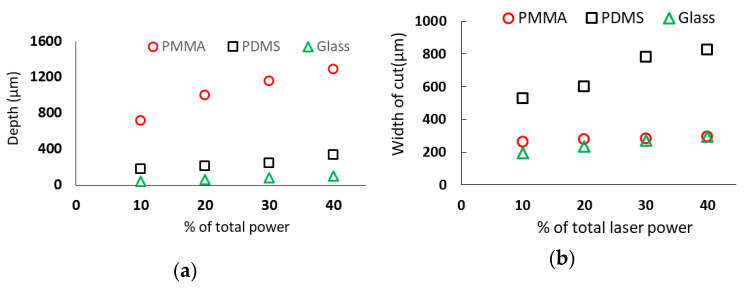
Effect of laser power on microchannel depth and width. The specimen was placed at the focal distance of the CO_2_ laser machining system. The laser system was set at single pass, scanning speed at 10 mm/s and current at 20 mA. The total laser power was 45W; (**a**) shows the depth and (**b**) width of the microchannels as a function of laser power.

The PMMA, PDMS and glass specimens were placed at the focal distance of 0.68 cm. The laser scanning speed, number of passes and current were set at 10 mm/s, 1 pass and 20 mA respectively while the laser power was varied as an increment of 10% to elucidate the effect of laser power on the laser-fabricated microchannel in these substrates. The total laser power was 45 watt. The minimum power utilized in this experiment was 4.5 W (i.e., 10% of total power) while the maximum power was 18 W (i.e., 40% of total laser power). The SEM images and measurement of PMMA microchannels for various laser power settings are shown in Figure 3. Figure 4 shows the effect of the laser power on substrate material channel depth and width. Both channel depth and width increased linearly with an increase in laser power. The increase in the percentage of total power increased the amount of heat used to ablate the surface. As a result, the depth, as well as the width of the cut increased. The slope of the best fit line based on the channel depths (not shown) in PMMA was the highest while that in the glass was the lowest. Hence it can be inferred that the depth increase rate was higher for the PMMA and glass while the width increase rate was higher for the PDMS. For instance, in the PDMS substrate using 4.5 W of laser power, the channel depth measured 182.3 µm while the width measured 528.3 µm. From these observations, it is clear that material properties especially laser absorptivity, laser penetration depth, thermophysical properties such as thermal conductivity, specific heat, thermal diffusivity, and melting temperature played important roles in controlling microchannel properties which are consistent with our previous computer modeling works [27] and others’ studies [17,35].

### 3.3. Effect of Laser Scanning Speed

In order to evaluate the effect of laser scanning speed on the microchannels, scanning speed was varied at 5 mm/s, 10 mm/s, 15 mm/s, 20 mm/s, 25 mm/s. 

**Figure 5:**
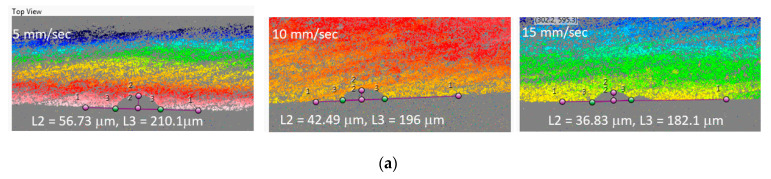

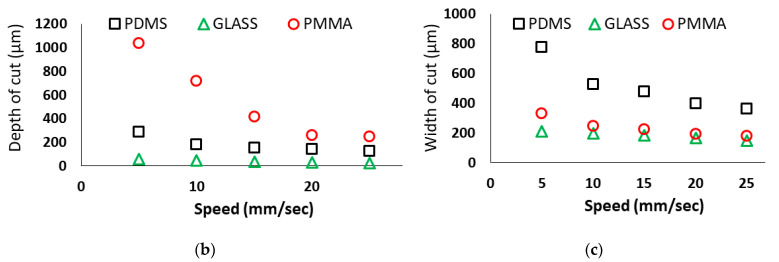
(**a**) 3D profilometer images and measurement of glass microchannel characteristics at various scanning speeds. The laser machined microchannel’s (**b**) depth and (**c**) width are shown as function of laser scanning speed. The specimen was placed at the focal distance of the CO_2_ laser engraving machine. The laser system was set at 10% i.e., 4.5 W, single pass and scanning speed at 10 mm/s.

The other laser parameters such as laser power at 4.5 W (10% of total laser power), focal distance at 0.68 cm and number of passes at 1 remained unchanged. Figure 5a shows the profilometer images and measurement of channel characteristics for the glass substrate for various speeds. The results in Figure 5 showed that the depth of the cut gradually decreased with an increase in speed in all three substrate materials and reached a minimum value of 127 µm for PDMS, 246 µm for PMMA and 22.4 µm for glass. PMMA had the highest depth of the cut (1035 µm) while glass had the lowest depth of 55.8 µm at 5 mm/s when compared to other two materials. Glass had the lowest depth of 55.8 µm when compared to other two materials. The scanning speed of the laser machine directly defines the exposure time of the laser beam on the substrate [27]. The lesser the speed, the more heat will be applied to the substrate. Thus, higher absorption of the laser energy leads to the increased depth of cut. Laser scanning speed not only affected the depth of the microchannel but also the width of the channel as seen in Figure 5c. The general trend of the scanning speed effect on channel width was similar to that of the cut depth. However, it is interesting to note that the rate of increase or decrease of width and depth was different for each material. The channel width in PMMA did not change much as compared to the cut depth. The channel depth drastically decreased with increased scanning speed. However, the rate of change in cut width for the PDMS channel was significant. In this particular investigation, PDMS had the highest cut width (777 µm) followed by PMMA (331 µm) while PMMA had the highest cut depth followed by PDMS. 

### 3.4. Effect of Number of Passes

**Figure 6:**
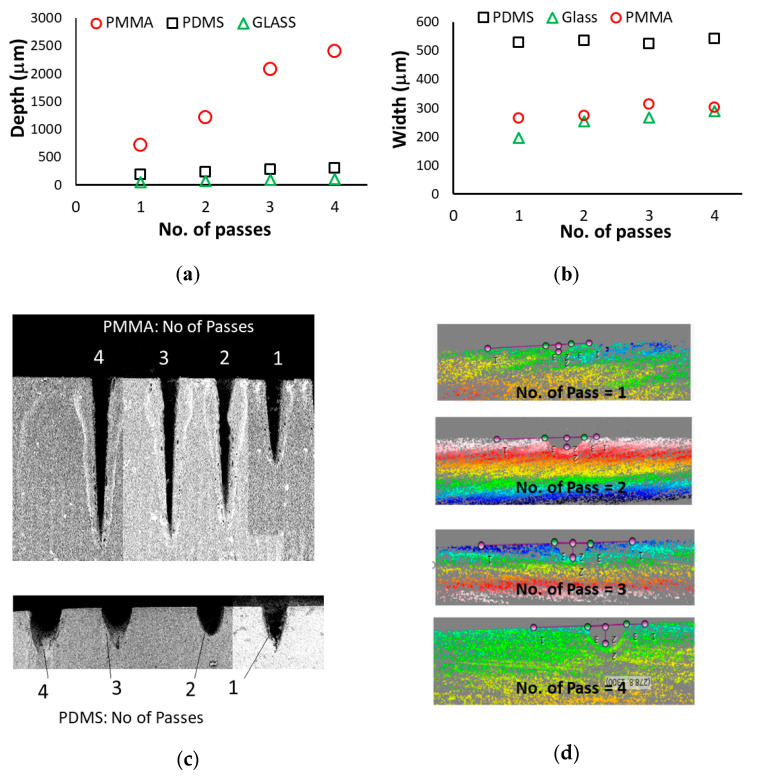
The effect of number of passes on the microchannel characteristics (**a**) depth, (**b**) width of the channels, (**c**) SEM images of PMMA and PDMS channels and (**d**) profilometer images of glass channels.

Figure 6 shows the effect of the number of passes on the laser-machined microchannels’ depth, width and the shape. The target specimens were placed at the focal distance of the focusing lens, and the laser power at 4.5 W and the scanning speed at 10 mm/s remained constant while the number of passes were varied. Figure 6a shows that the channel depth increased with the increasing number of passes. However, the rate of depth increase was not same in all materials. The rate of depth increase in the PMMA substrate was much higher than that of the PDMS and glass substrate. The maximum cut depth obtained for PMMA was 2410 µm whereas, for PDMS and glass, they were 298 µm and 108.7 µm respectively. In contrast, the rate of cut width increased much slower in all three types of substrates. The cut width for PDMS and PMMA almost remained constant with an increasing number of passes while it slightly increased in the glass substrate. PDMS had more cut width compared to PMMA and glass for the same number of passes. This observation again showed that the material types with relevant material properties such as thermal conductivity, specific heat, melting and decomposition temperatures have strongly influence the width and depth determination of the laser machined microchannels. It is relevant to note that one of the major challenges in the laser-machined microchannel is to control the channel shape and surface quality [17,26,36]. The tapered and skewed microchannels have very limited applications [36]. The circular or rectangular channel profiles are desirable. Use of multiple passes is one of the laser system parameters that can be used to control the shape of the channel [29]. Figure 6c,d shows the profiler images of channels in PMMA, PDMS and glass for varying passes. It is interesting to note that the number of passes in glass substrate made the channel more circular in cross-section; however, in PMMA, it made a sharper corner at the bottom. After the first pass, the distance between the target surface and the focusing lens changed due to material removal. This altered the strength i.e., intensity of the penetrating laser beam and increased its scattering (i.e., unfocused beams). Therefore, combining substrate material properties with dynamic changes in laser properties in multiple passes is a paramount interest of study in controlling laser-machined microchannel depth, width, shape and surface quality.

### 3.5. Fibroblasts Cell Adhesion Test

**Figure 7:**
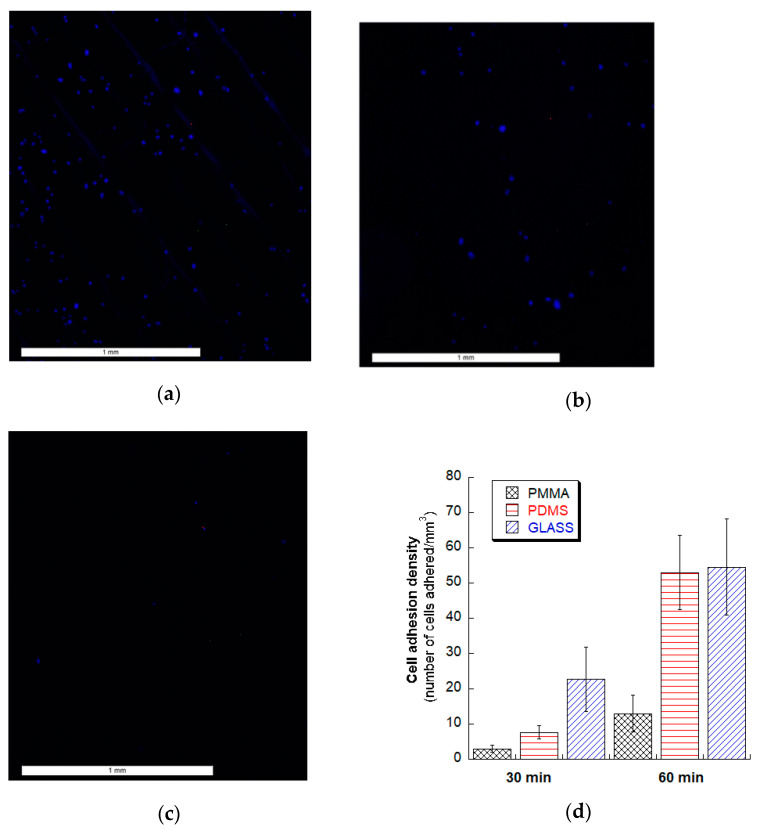
Human fibroblast cell adhesion test for 30-min incubation time for laser-machined microchannels on (**a**) glass, (**b**) PDMS, and (**c**) PMMA. (**d**) Quantified cell density on glass, PDMS and PMMA substrate with laser-machined microchannels for 30 min and 60 min incubation time.

Figure 7 shows human fibroblast cell adhesion on glass, PMMA and PDMS substrates. At 30 min of the incubation period, glass microchannels demonstrated the best cell adhesion density while PMMA channels had the least adhesion. We anticipate that the variation of surface roughness quality among the materials may have contributed to the resulting greater cell adhesion on the glass microchannels in addition to the surface chemistry of the substrates. It is notable that laser machined glass microchannels are susceptible to microcrack development [37] and these cracks can be a source of autofluorscence by trapping stains. When incubation time was doubled to 60 min, cell adhesion density on the PDMS channel approached similar cell density of glass microchannels. However, it was observed that for 2 h and more (up to 24 h) incubation time, cell adhesion density on all of these substrates with lasered microchannels did not vary. With this observation, PDMS and glass microchannels may be more suitable for microchannel based rapid biological applications such as microfluidic-based tissue engineering, in-vitro physiology studies, drug development, and cell separation assays or shear devices [38,39]. However, more experiments need to be conducted, along with surface chemistry and roughness analyses, to explain this observation and make a recommendation. 

## 4. Conclusions

This study presented an experimental parametric analysis of the CO_2_ laser machining of microchannels on the three most common microfluidic substrates—glass, polydimethylsiloxane (PDMS) and poly (methyl methacrylate) i.e., PMMA. The laser systems parameters—distance between the target object and focusing lens, laser power, scanning speed and number of passes—were varied one at a time to fabricate microchannels on these common substrates. The corresponding channel characteristics—depth, width and shape of the channel—were evaluated using a scanning electron microscope (SEM) and 3D profilometer. Human dermal fibroblast cells adhesion experiments on laser machined microchannels of these three substrates were also conducted. Results show that each of these laser system’s parameters had a significant role in determining depth, width and shape of the channel. However, the degree of impact of this laser system’s parameters were not the same. The cell adhesion test showed that human dermal fibroblast cells adhere faster on glass and PDMS channels compared to PMMA microchannels. Therefore, the type of substrate material i.e., material properties has a significant role in determining the characteristics of laser-machined microchannels as well as its biological applications. The results and discussions presented in this report can help to choose material-specific laser system parameters for microchannel fabrications using CO_2_ laser machining system. The following conclusions are made:

1. Depth and width of a microchannel increase with increasing the laser power in all three substrates. However, the rate of increase in width in PMMA and glass substrate is less pronounced than the increase in depth. For a given laser power, PMMA materials provided the deepest channel and PDMS substrates provided the widest channel.
2. For a given laser parameter, the depth of cut becomes maximum and the width of the cut becomes minimum when substrates are placed at the focal distance of the system in all three substrate materials.
3. Both the depth and width of the microchannel decrease with the increased laser scanning speed in all three substrates.
4. With the increase of the number of passes, the channel profile becomes more circular in glass and PDMS substrate while in PMMA it introduces a sharper corner at the bottom of the channel.
5. The cut width is more sensitive for PDMS while the cut depth is more sensitive in PMMA for different combinations of laser system parameters. On the other hand, glass is more delicate for laser machining because it breaks more easily and produces more broken edges at the machined microchannels.

## Figures and Tables

**Table 1:** Channel depth of laser machined microchannel for various distances between the substrate and focusing lens.

Focal Distance	0.3 cm					0.68 cm					1.06 cm				
/Substrates	S1	S2	S3	Avg	SE	S1	S2	S3	Avg	SE	S1	S2	S3	Avg	SE
PMMA (μm)	616	603	599	606	5.1	1030	972	998	1000	16.8	616	635	593	615	12.1
PDMS (μm)	162	162	162	162	0	220	188	227	212	12	169	156	169	165	4.33
Glass (μm)	28.3	28.3	28.3	28.3	0	62.3	62	63	62.6	0.22	28.5	28	28	28.4	0.05

## Data Availability

Essential data are contained within the article. The raw data are available on request from the corresponding author.
